# Surrogate model-based multi-objective Bayesian optimisation of porous acoustic barriers

**DOI:** 10.1007/s00366-025-02221-0

**Published:** 2025-10-24

**Authors:** Hassan Liravi, François-Xavier Bécot, Sakdirat Kaewunruen, Jelena Ninić

**Affiliations:** 1 Department of Engineering, University of Durham, Durham, DH13LE UK; 2 Matelys–Research Laboratory, 7 Rue des Maraîchers, 69120 Vaulx-en-Velin, France; 3 Department of Civil Engineering, School of Engineering, University of Birmingham, B15 2TT Birmingham, UK

**Keywords:** Surrogate model, Acoustic barrier, Multi-objective optimisation, Bayesian algorithm, Porous material

## Abstract

Many engineering challenges involve optimising multiple criteria that often represent conflicting targets, posing significant difficulties for standard methods like gradient-based algorithms. This complexity is especially important in the context of acoustic wave propagation, where noise barriers are designed to attenuate sound pressure level (SPL). Achieving optimal performance requires carefully balancing design factors such as shape and material selection with economic constraints, making the optimisation process both technically demanding and computationally intensive. This paper proposes the development of a noise prediction surrogate model for the multi-objective optimisation of acoustic barriers. This model is developed based on data set generated employing a two-dimensional singular boundary method. The optimisation process is conducted using a multi-objective Bayesian optimisation algorithm, which is applied to the problem of acoustic line source diffraction in the presence of a porous noise barrier. Two distinct barrier configurations are considered: a straight-walled barrier and a T-shaped barrier. With a view to reduce the SPL behind the noise barrier, the set of spanned parameters includes the SPL on the side of the barrier opposite to the source, barrier’s height, cap length of T-shaped barrier, porosity, tortuosity, and airflow resistivity of the material, integrating both microstructural and macrostructural aspects into the optimisation. Surface impedance boundary condition is used in the model to represent the dissipation at the surface of the noise barrier. The results demonstrate that the proposed optimisation framework enables efficient exploration of trade-offs to achieve an optimal barrier design that balances acoustic performance, material cost, and shape constraints.

## Introduction

The increasing level of noise pollution in urban environments, driven by rapid urbanisation and expanding transportation networks, has become a critical concern for modern cities. Prolonged exposure to high noise levels not only influences quality of life, but is also associated with severe consequences to human health [1, 2]. In this context, there is a growing emphasis on devising effective strategies to mitigate the noise that results from traffic. Scientific community and technology innovators are increasingly focusing on this matter, leading to the proposal of various practical solutions. Acoustic barriers have proven to be among the most practical and effective measures to mitigate environmental noise, significantly reducing noise propagation into sensitive areas. Their strategic implementation significantly enhances the acoustic comfort of residential zones, workplaces, and public spaces, contributing to sustainable urban development.

The design of acoustic barriers, however, is a complex and multi-faceted process. Key factors, based on the European standards, include acoustical characteristics, mechanical and safety characteristics, long-term performance and sustainability [3]. Achieving optimised designs that maximise noise attenuation while minimising material usage and construction costs is paramount [4]. Innovative computational methods for accurate prediction of sound behaviour around barriers as well as robust multi-objective optimisation strategies are crucial for addressing these complexities and facilitate the exploration of novel design solutions. Building on this foundation, the presented study aims to advance the field by proposing novel strategies for both the prediction and optimisation of barrier performance. The objective is to introduce new methodologies for designing noise barriers that are not only highly accurate and robust but also computationally efficient, setting a new benchmark for optimised acoustic barrier design.

Traditionally, numerical simulation techniques, including the finite element method (FEM) [5, 6] and boundary element method (BEM) [7–11] have been widely employed for evaluating the acoustic performance of barriers under various conditions. These methods allow for accurate modelling of sound propagation enabling detailed analysis of complex geometries and material behaviours; however, their computational efficiency is significantly influenced by the frequency of interest. The FEM and BEM typically require at least 10 degrees of freedom per wavelength in each spatial direction to produce the acceptable results [12]. This requirement leads to substantial computational costs, especially for large-scale acoustic problems, such as modelling acoustic barrier-related problems featuring complex structures. To address the limitations of conventional methods, recent research has explored numerical meshless methods, which do not rely on predefined domain or boundary meshes [13]. Among these methods, the method of fundamental solutions (MFS) and, more recently, the singular boundary method (SBM), which is of particular interest in this study, have emerged as promising boundary-type meshless techniques for simulating various engineering acoustic and vibration applications [14–17]. These methods offer computational efficiency together with numerical accuracy and robustness, making it particularly suited for analyses involving noise barriers. Within this context, investigations have discussed the potential of the MFS to deal with problems such as the assessment of the acoustic behaviour of thin T-shaped barriers [18] or even the evaluation of the effectiveness of metabarriers based on a periodic distribution of cylindrical elements [19]. However, it is noteworthy that the MFS may require the use of optimisation schemes to properly place its virtual sources or to incorporate modification strategies to accurately handle the geometrical complexity of noise barriers. The particularity of the SBM, compared to the MFS, is the placement of the virtual sources on the physical boundary, resulting in a more robust and effective methodology for addressing noise barrier-related challenges. The application of the SBM to this kind of problems began with the introduction of a two-and-a-half-dimensional (2.5D) SBM [20], which enabled the analysis of three-dimensional (3D) noise diffraction by thin T-shaped barriers. The SBM was subsequently extended to address various aspects of noise barrier design, including acoustic sensitivity analysis of barriers [21] and the optimisation of porous material thickness distributed on sound barriers [4, 22]. Further advancing the versatility of the SBM in this domain, a modified version of the method, hybridised with the MFS and referred to as the hybrid SBM-MFS, was specifically developed to address problems including geometric singularities in elastic media [23]. This hybrid approach was later adapted to tackle acoustic problems associated with noise barriers [24, 25], demonstrating its effectiveness in overcoming challenges inherent in practical engineering problems and extending the applicability of the SBM to acoustic barrier related problems featuring complex geometries. However, these methods remain computationally intensive, posing difficulties for optimisation when a large number of variables must be explored.

In parallel, optimisation techniques are increasingly utilised to enhance the design of noise barriers, aiming to identify optimal configurations that achieve desired noise reduction goals while adhering to practical constraints. These techniques are generally categorised as gradient-based and gradient-free methods. Gradient-based methods rely on sensitivity analysis involving derivatives of the objective function, offering faster convergence rates and finding extensive application in acoustic problem-solving [26–28]. In contrast, gradient-free methods, which use iterative trials to find optimal solutions without requiring sensitivity analysis, are slower in convergence but more versatile in handling complex problems characterised with multiple local minima. Among these, genetic algorithms (GA) and evolutionary algorithms (EA) (e.g. particle swarm optimisation (PSO)) stand out as prominent approaches. GA has been extensively applied to barrier optimisation challenges [29–32], but PSO has emerged as a preferred alternative due to its simplicity and efficiency. Unlike GA, PSO avoids intricate operations like crossover and mutation, operates directly with real numbers, and demands less computational memory [33].

Although GA and EA methods are effective in avoiding local minima, they rely on iterative solution that involves numerous evaluations of computationally expensive objective functions, which limits their applicability to real-world engineering problems [34]. This challenge highlights the need for a more efficient approach capable of balancing exploration and exploitation while minimising computational costs. Bayesian optimisation offers a compelling alternative in such scenarios [35–38]. Bayesian optimisation intelligently selects the next evaluation points based on probabilistic estimates of the objective function. This method not only reduces the number of required evaluations but also adapts to high-dimensional, noisy, and constrained problems [35], making it particularly well-suited for acoustic barrier problems or other scenarios with complex input-output coupling. Additionally, employing surrogate models, often generated based on supervised machine learning (ML) techniques [39], the optimisation precess can be realised in real time. Nevertheless, it is crucial to emphasise that the success of any surrogate-based search method is influenced by the accuracy and reliability of the surrogate model [40].

To address the challenge of complex and efficient multi-objective optimisation of porous acoustic barriers, this study introduces a novel framework combining the SBM with advanced surrogate and optimisation models. The SBM is used to predict and generate the data set to train the surrogate model. Surface impedance is incorporated into the numerical approach to represent porous materials. The optimisation component of this framework leverages computational efficiency of surrogate models, which are data-driven approximations of computationally expensive simulations. These supervised machine learning models include Radial Basis Function (RBF) [41], Artificial Neural Networks (ANNs) [42], and Random Forest (RF) [43]. Surrogate models enable efficient exploration of multi-dimensional design spaces by approximating objective functions and constraints, which are computationally intensive to evaluate directly. A key contribution of this paper is a comprehensive comparison of the efficiency and accuracy of these surrogate models to identify the most suitable approach for multi-objective Bayesian optimisation of acoustic barriers. Moreover, the optimisation process seeks to minimise acoustic pressure levels around the barriers by varying geometric and material parameters, such as barrier’s height, and the porosity, tortuosity, and airflow resistivity (AFR) of porous materials. The proposed framework evaluates the performance of straight-walled and T-shaped noise barrier configurations utilised in the context of a line source diffraction problem. To ensure the reliability of the surrogate models, a detailed accuracy analysis is conducted to evaluate their predictive capabilities in acoustic barrier design scenarios. This analysis enables the identification of the most efficient and accurate surrogate model for handling the complex multi-objective optimisation tasks required in acoustic barrier design. By integrating the computational strength of SBM with advanced surrogate modelling and optimisation techniques, this study aims to advance the design of acoustic barriers, providing optimised solutions that enhance noise mitigation while ensuring computational efficiency. This approach holds significant potential for improving urban noise management through innovative, data-driven design methodologies.

The remainder of this paper is organised as follows. Section 2 outlines the mathematical formulation of the 2D SBM approach for a half-space medium. Section 3 discusses the theoretical background of the surrogate models employed in this study. Section 4 provides a validation example to assess the accuracy of the numerical model. Section 5 conducts a parametric study based the selected geometrical and material parameters. Section 6 presents comparison examples to assess the accuracy of various surrogate models. In Sect. 7, single-objective and multi-objective optimisations of the porous acoustic barrier is carried out. Section 8 evaluates the performance of the optimised cases across the entire frequency range. In Sect. 9, a graphical user interface for predictions and optimisation has been implemented and explained. Finally, the main findings of this study are summarised in Sect. 10.

## Mathematical formulation

### Problem definition

**Fig. 1:**
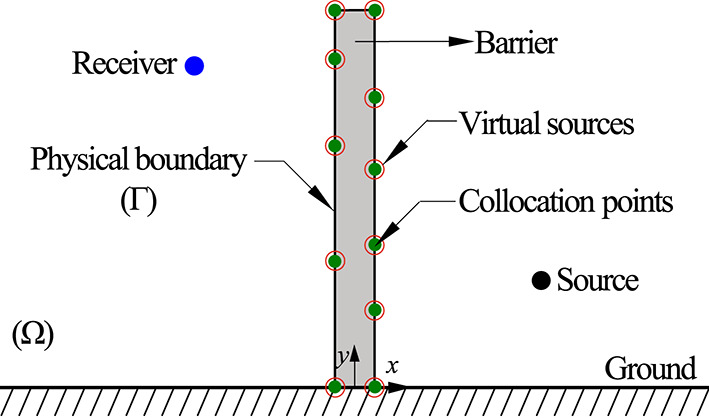
Schematic representation of the 2D SBM approach for a straight-walled barrier, including geometry and distributions of collocation points and virtual sources. Collocation points are highlighted by green solid dots, while virtual sources are indicated by red circles. The line source and receiver are represented by black and blue circles.

The problem under consideration is the propagation of acoustic waves in a homogeneous isotropic medium $\Omega$, in the presence of a barrier situated above an infinite planar ground surface, as illustrated in Fig. 1. For simplicity and practicality, only the 2D case is analysed, as it sufficiently captures propagation paths normal to the barrier. As demonstrated in previous studies [7], the insertion loss calculations for 2D and 3D noise barriers yield equivalent results when considering normal sound wave propagation. In this configuration, the acoustic field is generated by an external harmonic line source positioned at ${\mathbf {x_0}}=\{x_0, y_0\}^\text {T}$. The resulting pressure field in the frequency domain can be mathematically modelled using the 2D Helmholtz equation as

1 $$\begin{aligned} \nabla ^2 p \left( {\textbf{x}}\right) + k^2\, p\left( {\textbf{x}}\right) = 0 \quad \text {for} \quad {\textbf{x}}\in \Omega , \end{aligned}$$

where $p(\textbf{x})$ represents the acoustic pressure at a generic point ${\textbf{x}}=\{ x, y\}^\text {T}$ inside the domain, *k* is the acoustic wavenumber and it is equal to $\omega /c$, $\omega$ is the angular frequency and *c* is the sound wave speed in the medium.

For the specified problem, half-space fundamental solutions are used to model the unbounded domain where the ground is treated as an infinite plane upon which the sound waves are perfectly reflected. Half-space fundamental solutions can be derived using the image-source technique, resulting in the following solutions for the acoustic pressure and particle velocity

2 $$\begin{aligned} G(x,x_0,k) = \displaystyle \frac{\text {i}}{4} \big [H^{1}_{0} \left( kr\right) + H^{1}_{0} \left( kr^{\prime }\right) \big ], \end{aligned}$$

3 $$\begin{aligned} \displaystyle \frac{\partial G (x,x_0,k)}{\partial \textbf{n}_x} = \displaystyle \frac{\text {-i}{k}}{4} \big [H^{1}_{1} \left( kr\right) + H^{1}_{1} \left( kr^{\prime }\right) \big ]\displaystyle \frac{\partial r}{\partial \textbf{n}_x}, \end{aligned}$$

respectively. In these equations, $H^{1}_{0}$ and $H^{1}_{1}$ are the Hankel functions of the first kind of order zero and one, respectively, $r=\sqrt{(x-x_0)^2 + (y-y_0)^2}$ and $r^\prime =\sqrt{(x-x_0)^2 + (y+y_0)^2}$ and $\textbf{n}_x$ stands for an arbitrary unit vector that represents the direction along which the particle velocity is calculated. Furthermore, the incident wave field at the barrier due to the line source at any arbitrary point $\textbf{x}$ is given by

4 $$\begin{aligned} p_{inc}(x,x_0) = \displaystyle \frac{\text {i}}{4} \big [H^{1}_{0} \left( kr\right) + H^{1}_{0} \left( kr^{\prime }\right) \big ], \end{aligned}$$

and the Neumann and Robin boundary conditions are represented as

5 $$\begin{aligned} \begin{aligned}&\frac{\partial {{p}_\text {tot} ({\textbf{x}})}}{\partial {\textbf{n}_b}} = \frac{\partial {{p}_\text {inc} ({\textbf{x}})}}{\partial {\textbf{n}_b}} + \frac{\partial {{p}_\text {dif} ({\textbf{x}})}}{\partial {\textbf{n}_b}} = 0 \quad \text {for} \quad {\textbf{x}}\in \mathrm {\Gamma }, \\&\frac{\partial {{p}_\text {dif} ({\textbf{x}})}}{{\partial {\textbf{n}_b}}} = - \frac{\partial {{p}_\text {inc} ({\textbf{x}})}}{\partial {\textbf{n}_b}} = \text {i} \rho \omega {v}_{b} ({\textbf{x}}) \quad \text {for} \quad {\textbf{x}}\in \mathrm {\Gamma }, \end{aligned} \end{aligned}$$

6 $$\begin{aligned} Z_s = \displaystyle \frac{p_b ({\textbf{x}})}{v_b ({\textbf{x}})} \quad \text {for} \quad {\textbf{x}}\in \mathrm {\Gamma }, \end{aligned}$$

respectively, where ${p}_\text {tot}$ and ${p}_\text {dif}$ denote the total and diffracted pressure fields, respectively, $p_{b}$ and $v_b$ are the prescribed pressure and normal velocity fields at the boundary, respectively, $\textbf{n}_b$ is the unit outward normal to the physical boundary at the point $\textbf{x}$, $\rho$ is the medium density, $\text {i}=\sqrt{-1}$ and $Z_s$ represents the surface impedance parameter. Note that it is presumed that sound propagation is not accounted for within the barrier.

### 2D SBM formulation for acoustic half-space medium

The SBM is employed to solve the above-stated problem. In this approach, *N* collocation points are systematically positioned along the physical boundary ($\Gamma$), coinciding with the source points, as depicted in Figure 1. This placement obviates the need for domain discretisation, which is a defining feature of this meshless methodology. The solution to the Helmholtz equation, Eq. (1), is approximated as a linear combination of fundamental solutions, leveraging the boundary collocation technique to enforce boundary conditions effectively, as

7 $$\begin{aligned} p ({\textbf{s}}_m)=\alpha _m G_{mm}+\sum _{j=1, j \ne m}^{N} \alpha _j\, G({\textbf{s}}_m,{\textbf{s}}_j, k), \quad {\textbf{s}}_m \in \, \Gamma , \end{aligned}$$

8 $$\begin{aligned} {\text {i} \rho \omega } v ({\textbf{s}}_m) = \alpha _m Q_{mm} + \sum _{j=1, j \ne m}^{N} \alpha _j\, \frac{\partial G({\textbf{s}}_m,{\textbf{s}}_j, k)}{\partial \textbf{n}_b} , \quad {\textbf{s}}_m \in \, \Gamma , \end{aligned}$$

9 $$\begin{aligned}&\text {i} \rho \omega v ({\textbf{s}}_m) + \text {i} Z_s p ({\textbf{s}}_m) = \alpha _m \left( \text {i} Z_s G_{mm} + Q_{mm} \right) + \nonumber \\ &\sum _{j=1, j \ne m}^{N} \alpha _j\, \left[ \text {i} Z_s G ({\textbf{s}}_m,{\textbf{s}}_j, k_a) + \frac{\partial G({\textbf{s}}_m,{\textbf{s}}_j, k)}{\partial \textbf{n}_b} \right] , \quad {\textbf{s}}_m \in \, \mathrm {\Gamma }, \end{aligned}$$

where $G_{mm}$ and $Q_{mm}$ are defined as the origin intensity factors (OIFs) of the fundamental solutions of the Helmholtz equation and $\alpha _j\,(j=1,2,...,N)$ are the unknown source strengths.

By utilising Eqs. (8) and (9) and aligning them with the boundary conditions defined in Eqs. (5) and (6), a linear system of equations is formulated as

10 $$\begin{aligned} A \alpha = b, \end{aligned}$$

where *A* is the coefficient matrix, *b* corresponds to the boundary condition vector, and $\alpha$ comprises the unknown coefficients to be determined. The matrix *A* and vector *b* are fully determined through the specified boundary conditions. Once the coefficients $\alpha$ are resolved, the sound pressure *p*(*x*) at any arbitrary point within the domain can subsequently be evaluated as

11 $$\begin{aligned} p ({\textbf{x}})=\sum _{j=1}^{N} \alpha _j\, G({\textbf{x}},{\textbf{s}}_j, k), \quad {\textbf{x}} \in \, \Omega . \end{aligned}$$

To derive the origin intensity factors (OIFs) associated with the SBM, this study employs a desingularisation strategy based on the subtracting and adding-back technique. This method, as elaborated in several studies focused on SBM [44, 45], ensures accurate treatment of singularities in the fundamental solutions. The resulting approach leads to the following formulations

12 $$\begin{aligned} G_{mm} = \frac{\text {i}}{4} - \frac{1}{2\pi } \left( \ln \left( \frac{L_m}{2\pi }\right) +\ln \left( \frac{k}{2}\right) +\gamma \right) , \end{aligned}$$

13 $$\begin{aligned} Q_{mm} = \displaystyle \frac{1}{L_m}\, \left( 1-\sum _{j=1,j\ne m}^{N} L_j\, \frac{\partial {G_L ({\textbf{s}}_m,{\textbf{s}}_j)}}{\partial {\textbf{n}_{s_{j}}}} \right) , \end{aligned}$$

where $L_j$ is the half length of the curve between the source points $s_{j-1}$ and $s_{j+1}$ on the physical boundary, $\gamma$ denotes the Euler-Mascheroni constant and $G_L =-\ln (r)/ {2\pi }$ represents the fundamental solution of Laplace equation.

### Surface impedance parameter

Considering the potential utilisation of noise-absorbing materials combined with the barrier, this study specifically investigates the influence of porous material on the acoustic response of the barrier. This can be introduced by imposing acoustic absorption boundary conditions (Robin boundary condition) within the framework of the developed 2D SBM approach, as previously described in Eq. (6). In this equation, $Z_s$ is the surface impedance parameter. For a typical porous material considered in this paper, characterised by a rigid skeleton saturated with air at rest, and for a layer of thickness *l*, the surface impedance is given by [46, 47].

14 $$\begin{aligned} Z_s = Z_c \coth \left( -\text {i}\,k_c\,l \right) , \end{aligned}$$

where $Z_c$ is the characteristic impedance of the porous material and $k_c$ is the complex wave number of the porous material. Thus, the acoustical behaviour of a rigid-frame porous material is completely characterised by specification of its characteristic impedance $Z_c$ and its complex wave number $k_c$. In developing theoretical models to predict these quantities, it is convenient to treat viscous and thermal effects within the pores separately; these effects are described by complex dynamic density $\rho _g$ and bulk modulus $K_g$ functions, respectively, as [46]

15 $$\begin{aligned} k_c = \omega \left[ \rho _g (\omega )/K_g(\omega )\right] ^{1/2}, \end{aligned}$$

16 $$\begin{aligned} Z_c = \left[ \rho _g (\omega )/K_g(\omega )\right] ^{1/2}/\Omega , \end{aligned}$$

with $\Omega$ being the porosity of the material. The detailed information of the theoretical model considered for the porous materials along with calculation of the porosity and tortuosity can be found in [46].

It should be mentioned that the calculation of the surface impedance parameter has been performed using AlphaCell^®^ software [48], developed by the Matelys Research Lab.

## Theoretical background of surrogate modelling and optimisation algorithms

In recent years, ML has achieved remarkable advancements in the field of acoustics, often outperforming traditional signal processing techniques. Due to the evident need for rapid computation, robustness, and adaptability in all areas of complex numerical simulations, surrogate models, also known as metamodels, have often been used over the last three decades to substitute numerical simulations. Simulation surrogates establish the dependency between simulation inputs and outputs, learning multiple relationships between different parameters and mapping them to a given output. Different methods are used to provide compact representations of simulation models, e.g., regression models [49], Neural Networks (NNs) [50], RBFs [51], support vector regression [52], Kriging or spatial correlation models [53, 54], response surfaces [55], game theory models [56], proper orthogonal decomposition [57], etc. These models are employed to simplify and interpret simulation models [58], conduct sensitivity and what-if analyses, and optimise the simulation output [59]. Yet, the performance and suitability of different methods for generating surrogates heavily depend on the application. Hence, a rigorous evaluation of various methods for establishing reliable surrogates is an essential task.

### Regression models

Regression models are designed to predict continuous responses, such as data trends commonly utilised in surrogate modelling. This section introduces the regression models employed in this study for predicting acoustic wave propagation. These models were selected for their distinct features and capabilities. RF is robust to noise, even when dealing with a very large number of independent variables [60], RBF networks offer smooth interpolation in multi-dimensional spaces, making them particularly suitable for unstructured data where polynomial or spline interpolation is often infeasible [61], and ANN is well-suited for approximating non-linear, high-dimensional partial differential equations [62]. The chosen models thus provide a balanced trade-off between predictive accuracy, generalisation capability, and computational efficiency for the problem under consideration.

#### Artificial Neural Network

Surrogate models inspired by biological functions, particularly those found in the brain and nervous system, are known as ANNs. The structure of ANNs is typically represented as a system of interconnected neurons. An ANN surrogate model can be expressed as [63]:

17 $$\begin{aligned} {\hat{f}}(x) = \sum _{i=1}^N w_i \psi \left( \textbf{W}_i \cdot x + b_i\right) , \end{aligned}$$

where $w_i$ and $b_i$ are the weights and bias terms associated with the $i$-th neuron, $\psi$ represents the activation function, and $\textbf{W}_i$ denotes the weight vector of the $i$-th neuron corresponding to the input features. For this study, 10 hidden neurons are used in conjunction with Levenberg-Marquardt training for the optimisation of the weights and biases.

#### Random Forest

RF is a specialised predictor, primarily composed of an ensemble of randomised decision trees, denoted as $\{f(x; \Theta ^{(i)}_t, R_n)\}_{1 \le i \le T}$. The sequence $\{\Theta ^{(i)}_t\}_{1 \le i \le T}$ encapsulates the random variables $\Theta$ that govern the probabilistic mechanism underlying the construction of each tree. For a finite number of trees, $T$, the RF estimate can be expressed as [38, 64]:

18 $$\begin{aligned} f_T\left( X; \Theta _{1_t}, \Theta _{2_t}, \dots , \Theta _{T_t}R_n\right) := \frac{1}{T} \sum _{i=1}^{T} f\left( X; \Theta _{i_t}R_n\right) \end{aligned}$$

In this study, the RF model uses a least squares boosting algorithm with 100 boosting iterations and a learning rate of 0.05. The least square algorithm selects several bootstrap samples, $(R_n^{\Theta _{1_t}}, ...,R_n^{\Theta _{T_t}})$ containing *n* observations, and applies the previous tree-based decision algorithm to these samples to construct a collection of *T* prediction trees. Additionally, the minimum number of samples required in a leaf node of the tree is set to 5, and the maximum number of splits allowed in a tree is set to 20.

#### Radial basis function

RBFs approximate a complex design landscape by combining a weighted sum of simple predefined functions. Given $n$ sample points, the RBF surrogate model is formulated as [65]:

19 $$\begin{aligned} {\hat{f}}(x) = \sum _{i=1}^N w_i \psi \left( \left\| x - x^{(i)}\right\| ^2\right) , \end{aligned}$$

where $w_i$ represents the weights, determined through the least-squares method, and $\psi$ is the selected basis function. In this study, the maximum number of neurons and the number of neurons to be added between layers are set to 20 and 10, respectively. It is worth noting that, unlike the regression models introduced earlier, RBFs are treated as an interpolation technique.

### Multi-objective Bayesian optimisation

#### Bayesian optimisation

Bayesian optimisation (BO) is a powerful technique for the optimisation of acoustic barriers. It is considered as a low-cost global optimisation tool for design problems having expensive black-box objective functions. The general idea of the BO is to emulate an expensive unknown design space and find the local and global optimal locations while reducing the cost of function evaluation from expensive high-fidelity models. BO adopts a Bayesian perspective and assumes that there is a prior on the function; typically, a surrogate model prior is used. The overall BO approach has two major components: a predictor (in our case a surrogate model) and the acquisition function.

The ultimate goal of BO is to determine the global minimiser (or maximiser) of an unknown objective function, *f*, as [35]:

20 $$\begin{aligned} x^* = \underset{x \in {\mathcal {X}}}{\arg \min } \; {\mathbb {E}}_{p(f \mid {\mathcal {D}})} \left[ f(x) \right] , \end{aligned}$$

where ${\mathcal {X}}$ is the domain of *x*, ${\mathcal {D}} = \{(x_i, f(x_i))\}_{i=1}^{n}$ is the dataset of observations, and $p(f \mid {\mathcal {D}})$ denotes the posterior distribution over the objective function given the data.

In practice, BO does not directly minimise this expectation. Instead, it proceeds iteratively by defining and maximising an acquisition function $\alpha (x;{\mathcal {D}})$, which quantifies the utility of evaluating the objective at *x*. At iteration *t*, the next candidate point is chosen as

21 $$\begin{aligned} x_{t+1} = \underset{x \in {\mathcal {X}}}{\arg \max } \; \alpha \left( x; {\mathcal {D}}_t\right) , \end{aligned}$$

where ${\mathcal {D}}_t$ is the dataset after *t* evaluations.

In this work, BO is implemented in Matlab. The optimisation was carried out over a set of design parameters, including the height and cap length of the barrier (for the T-shaped barrier), as well as the porosity, AFR, and tortuosity of the porous material. The acquisition function was chosen as the *expected-improvement-per-second-plus*, a variant of the Expected Improvement criterion that considers both solution quality and computational efficiency. BO sequentially explores the design space by selecting the next most promising point based on the surrogate model. The maximum number of function evaluations was restricted to 200. Furthermore, the objective function was treated as stochastic to account for variability in the model outputs.

#### Multi-objective optimisation

The numerical optimisation problems can be classified on the basis of number of objective functions as Single-Objective Optimisation (SOO) and Multi-Objective Optimisation (MOO). MOO is the extension of SOO with having more than one objective as

22 $$\begin{aligned} \text {min} \; f(\textbf{x}) = \left[ f_1(\textbf{x}), f_2(\textbf{x}),..., f_q(\textbf{x})\right] , \quad \textbf{x} \in {\mathcal {X}}. \end{aligned}$$

It is obvious that SOO is relatively simpler and has a lower computational cost. However, in practical problems, it is rare to encounter a purely single-objective problem; hence, significant attention has been given to MOO methods. The key challenge in any MOO problem is determining the optimal design decisions based on user-defined preferences for multiple objectives. Optimal solutions at different trade-offs between objectives form a set of non-dominated solutions. Methods for solving MOO problems can be classified into a priori and a posteriori approaches. The most fundamental *a priori* approach is the weighted-sum method [66], in which all objectives are combined into a single weighted objective function. This method has also been employed in the present study.

Multi-objective Bayesian optimisation (MOBO) extends BO to the MOO setting by employing surrogate models and acquisition functions tailored to multiple objectives. At each iteration, the next evaluation point is selected by maximising a multi-objective acquisition function, e.g.

23 $$\begin{aligned} x_{t+1} = \underset{x \in {\mathcal {X}}}{\arg \max } \; \alpha _{\textrm{MOBO}}\left( x; {\mathcal {D}}_t\right) , \end{aligned}$$

where $\alpha _{\textrm{MOBO}}$ in the above equation is designed to encourage exploration of Pareto-efficient regions.

## Validation of the numerical model

Although the SBM has been thoroughly evaluated in previous studies [16, 24], a validation case is presented in this section to further demonstrate its accuracy. To this end, Example 2 from the study conducted by Wei et al. [67] is replicated. In this example, a rigid T-shaped barrier with a height of 2.05 m, a thickness of 0.05 m, and a cap length of 0.35 m is placed on a rigid ground surface. Two point sources are located at coordinates $(0.2,\,0.3)$ m and $(1.2,\,0.3)$ m, respectively. A total of 2500 evaluation points are uniformly distributed within a rectangular domain defined by a horizontal range of $(-2,\,2)$ m and a vertical range of $(0,\,4)$ m. For all computations, the acoustic medium is assumed to be air, with a density of $\rho = 1.225~\mathrm {kg/m^3}$ and a corresponding sound speed of $c = 340~\mathrm {m/s}$.

**Fig. 2:**
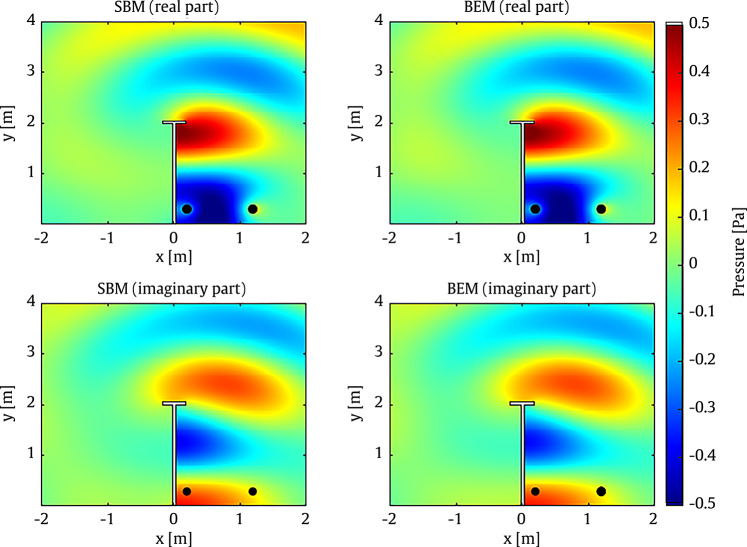
Pressure calculated by the SBM (left) and BEM (right) in 2D. The positions of the two line sources are indicated using solid black circles

The results obtained using the 2D SBM are compared against those from the 2D BEM. The BEM results were computed using the OpenBEM software [68], employing linear boundary elements. Both the real and imaginary parts of the pressure field within the domain are presented. As illustrated in Fig. 2, the SBM yields highly accurate results, showing excellent agreement with those obtained from the BEM. It should be noted that a previous study demonstrated that the 2D SBM is generally more accurate than the 2D BEM while requiring significantly less computation time [16]. Specifically, for the current validation case performed using the University of Birmingham’s primary computing facility, BlueBEAR, the 2D SBM was shown to be three times faster than the 2D BEM. Additionally, the average difference between the results of the 2D SBM and the reference solution in this comparison is approximately 2% across all evaluation points considered. This comparison highlights the validity of the SBM approach as a reliable foundation for both the surrogate model and the optimisation algorithm.

## Parametric study of porous acoustic barrier

**Fig. 3:**
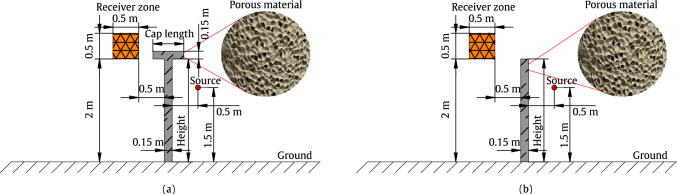
Considered geometrical variables for the T-shaped (**a**) and straight-walled (**b**) barrier types

**Fig. 4:**
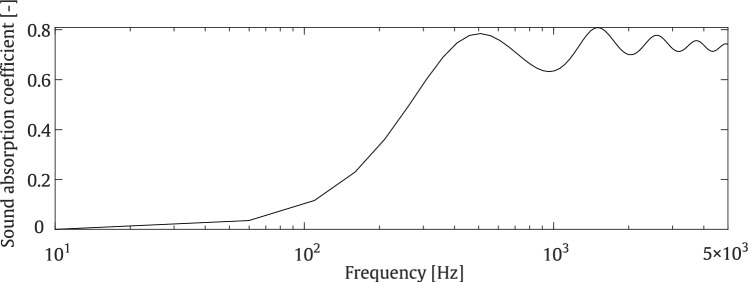
Sound absorption coefficient plot of the considered porous material in the parametric study within the frequency range of 0–5000 Hz

**Fig. 5:**
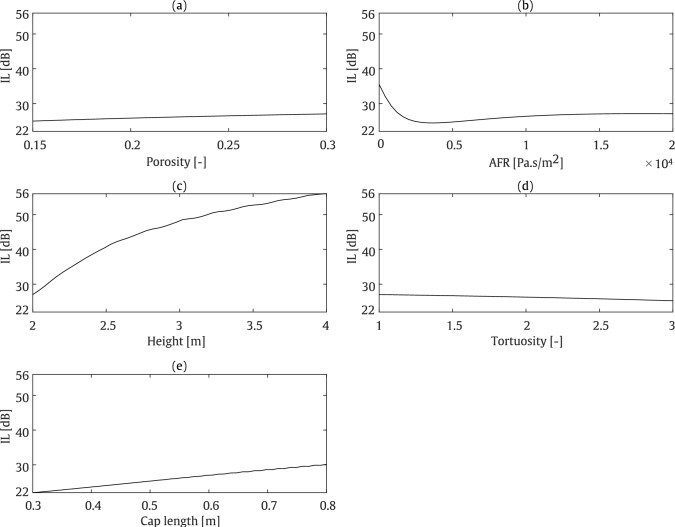
Parametric study of the influence of the considered parameters on the average pressure at receiver zone and at a frequency of 800 Hz

To gain a deeper understanding on the influence of the considered parameters on the pressure behind the barrier, a parametric study is conducted on various variables, including the height and cap length of the T-shaped barrier, as well as the porosity, tortuosity, and AFR of the porous material, all varied within a physically meaningful range. The considered geometrical parameters are visualised in Fig. 3. The range and step size for the variation of each parameter in the parametric study are provided in Table 1. Once the upper and lower limits of the considered parameters are defined, the impact of the selected parameters can be analysed in subsequent steps. To achieve this, a reference model with predefined variables is first established. In the reference model, the barrier height and cap length are assumed to be 2 m and 0.6 m, respectively, while the porosity, tortuosity, and AFR of the porous material are set to 0.3, 1, and 15,000 $\mathrm {Pa \cdot s/m^2}$, respectively. The sound absorption coefficient associated with the considered porous material is computed using AlphaCell^®^ software and presented in Fig. 4, within the frequency range of 0–5000 Hz. Each variable is then evaluated individually, with the other parameters remaining fixed at their reference values. This one-at-a-time design ensures that only the target variable is varied within its defined limits, as shown in Table 1. For this parametric study, a line source is positioned at (0.5, 1.5) m, while the receiver points are located in a square zone, as shown in Fig. 3, on the left side of the barrier, opposite to where the source is located. With this position, the sound pressure levels in the targeted zone result from diffraction exclusively. Figure 5 illustrates the impact of various parameters on the pressure values. The results are compared in terms of insertion loss (IL) formulated as:

24 $$\begin{aligned} \text {IL} = -20 \log _{10} \frac{|p_{\text {tot}}|}{|p_{\text {inc}}|} \end{aligned}$$

where $p_{\text {tot}}$ is the total pressure at an arbitrary point, and $p_{\text {inc}}$ represents the incident acoustic pressure generated by the source above the ground in the absence of the barrier, as previously defined in Eq. (4).

As illustrated in Fig. 5, this parametric study reveals that porosity and tortuosity have the lowest impact on the IL within the specified ranges of variation, with changes of approximately 2 dB. The geometrical parameters of the barrier, including height and cap length, are the most influential parameters, with variations of around 25 dB and 8 dB, respectively. Furthermore, an inverse trend is observed between porosity, barrier height, and cap length in relation to IL, whereas tortuosity and AFR exhibit an inverse relationship with IL, particularly when AFR falls below 2500 $\mathrm {Pa \cdot s/m^2}$, where significant changes become apparent.

**Table 1:** Ranges of variation of each parameter for the surrogate model.

No.	Variable	Range
1	Height [m]	From 2 to 4
2	Cap length (T-shaped) [m]	From 0.3 to 0.8
3	Tortuosity [–]	From 1 to 3
4	Porosity [–]	From 0.15 to 0.3
5	Airflow resistivity [Pa.s/m^2^]	From 0 to 20000

## Surrogate models for acoustic barriers

Surrogate models, serving as instant prediction tools, bridge the gap between computationally expensive numerical models and resource-intensive multi-objective optimisation. In this section, the previously introduced surrogate models are implemented and executed to evaluate their performance. In the parametric study, the influence of the barrier’s height and cap length, material porosity, tortuosity, and AFR is evaluated. All five parameters are treated as input variables in the learning database. It is essential to represent these parameters in the learning database in a balanced and systematic manner to avoid misleading results, overestimations, or underestimations. To achieve this, the Latin Hypercube Sampling (LHS) approach is employed to generate the input parameters. The primary objective of using the LHS technique is to reduce the number of simulations required while ensuring reasonable and reliable results [69]. Data pre-processing ensures that all variables are suitably distributed during the training process, as incorporating input parameters with differing distributions can cause the model to exhibit bias towards features with larger values and variances. A total of 250 samples is used to generate the dataset, as increasing this number did not significantly improve the accuracy of the trained model. The dataset is divided into training and test sets, with an 80% training and 20% test split. Furthermore, pre-processing enhances the speed of the learning process. In this study, input parameters have been preprocessed employing normalisation, where the input data are re-scaled into a range between 0 and 1 using the equation below:

25 $$\begin{aligned} X_{normalised} = \frac{X - X_{\textrm{min}}}{X_{\text {max}} - X_{\textrm{min}}}, \end{aligned}$$

where $X_{\textrm{min}}$ and $X_{\textrm{max}}$ represent the minimum and maximum value of input parameters, respectively. The surrogate models are generated to analyse the 2D problem of source diffraction using two computational examples: a straight-walled acoustic barrier and a T-shaped acoustic barrier, both filled with porous materials. In all numerical examples presented in this study, Robin boundary conditions are imposed on the boundary of the barrier using Eq. 9. Two frequencies, 200 Hz and 800 Hz, are selected for the error analysis in this study because the sound absorption levels differ significantly at these frequencies, as shown earlier in Fig. 4. For all calculation examples, a grid of 2500 receiver points is distributed on both sides of the barrier, enclosed by a reference plane defined by four boundary points: (– 1.5, 0) m, (– 1.5, 6) m, (1.5, 6) m, and (1.5, 0) m. The locations of the source and receiver points were previously demonstrated in Fig. 3. In the following, the three surrogate models are compared in terms of accuracy and computational efficiency. It should be noted that 10 nodes per wavelength, considering the frequency of interest, are assumed on the boundary of the barrier to ensure accurate results provided by the SBM approach.

### Comparison of accuracy

design The aim of this section is to evaluate the accuracy of the considered surrogate models. The results are first presented in terms of Sound Pressure Level (SPL), calculated using Eq. (26):

26 $$\begin{aligned} \textrm{SPL} = 20 \log _{10} \big |\frac{p}{p_\text {ref}}\big |, \end{aligned}$$

where $p_\text {ref}$ is reference pressure and is considered to be 20 $\mu$Pa.

Furthermore, the accuracy of the proposed surrogate models is assessed in the basis of the root mean square error (RMSE), which is computed using a set of $N_t$ test points located in the field and it can be defined as

27 $$\begin{aligned} \textbf{RMSE} = \sqrt{ \frac{ \displaystyle \sum _{k=1}^{N_t} \left| (\text {SPL})_s(\textbf{x}_k) - (\text {SPL})_r(\textbf{x}_k) \right| ^2}{ \displaystyle \sum _{k=1}^{N_t} \left| (\text {SPL})_r(\textbf{x}_k) \right| ^2} }, \end{aligned}$$

where the subscripts *s* and *r* represent the computed SPL at test point $\textbf{x}_k$ by each surrogate model method and the reference approach, respectively.

#### Straight-walled barrier

**Fig. 6:**
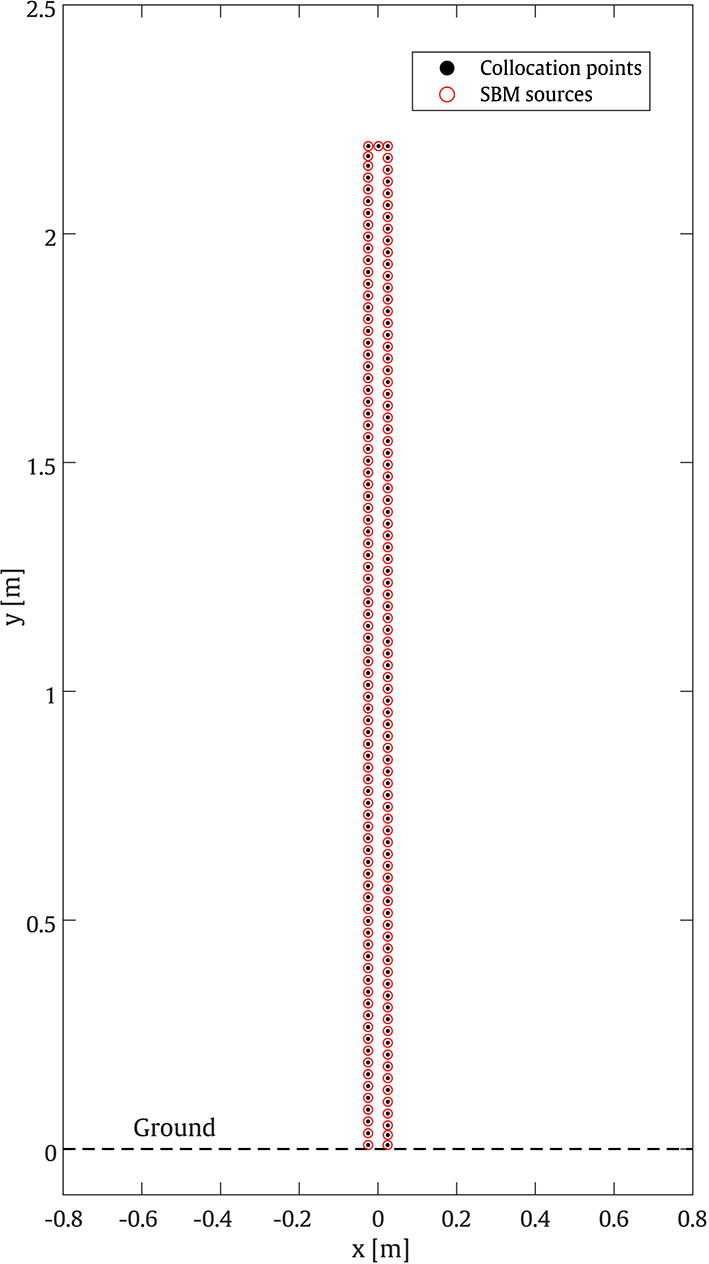
Configuration of the collocation points and sources used to adopt the 2D SBM for the considered straight-walled barrier problem

In this example, the SPL is calculated for a straight-walled case filled with porous material. For this case, the parameters are chosen from the validation dataset, where the height of the barrier is set to 2.2 m, while the porosity, tortuosity, and AFR of the porous material are defined as 0.23, 2.4, and 3560 $\mathrm {Pa \cdot s/m^2}$, respectively. In implementing the 2D SBM method, which serves as the ground truth, 172 uniformly distributed collocation points are placed along the boundary of the barrier, as shown in Fig. 6.

**Fig. 7:**
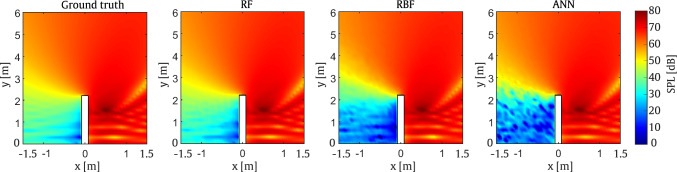
Comparison of SPL predictions from the surrogate models and the SBM at 800 Hz for the straight-walled barrier

The results presented in Fig. 7 demonstrate that all three surrogate models are capable of predicting accurate results when compared with the ground truth obtained from the 2D SBM approach. Overall, it can be observed that the RF model is slightly more accurate than the other approaches, particularly when the SPL on the left side of the barrier, opposite to the line source placement, is targeted. Specifically, the average RMSE of the RF method in the entire area is approximately 1.3%. Furthermore, a clear SPL attenuation is observed behind the barrier in all models, mainly at positions where only diffracted waves are received, as expected.

**Fig. 8:**
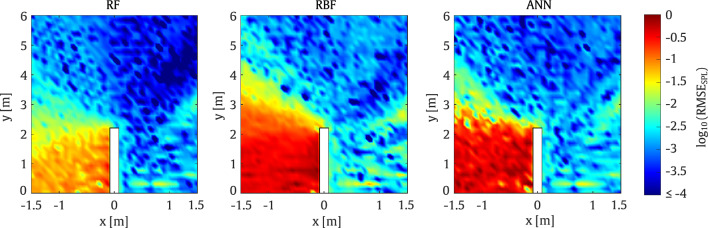
RMSE obtained by the surrogate models taking the SBM as the reference for a set of evaluation points at frequency of 800 Hz for the straight-walled barrier

The RMSE values presented in Fig. 8 confirm the trends observed in Fig. 7. Generally, it can be seen that all three methods are accurate. The RF model exhibits higher accuracy in most positions, with errors of up to 3% observed at specific evaluation points behind the barrier.

#### T-shaped barrier

**Fig. 9:**
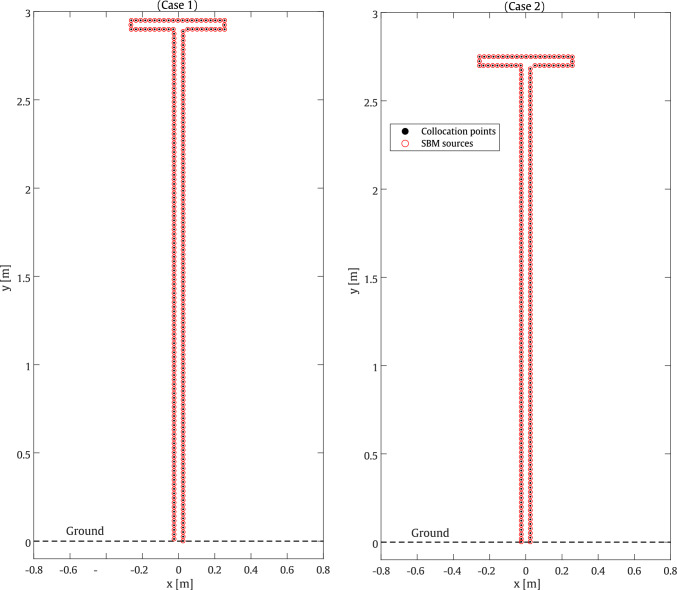
Configuration of the collocation points and sources used to adopt the 2D SBM for the considered T-shaped barrier problem

In the second example, the performance of the developed surrogate models is evaluated within the framework of a line source diffraction problem in the presence of a T-shaped barrier. The barrier is equipped with porous materials and features a cap with a length of $0.56 \, \textrm{m}$ in the first case and $0.52 \, \textrm{m}$ in the second case. The evaluation is carried out for two random test cases including parameters as shown in Table 2. It should be noted that the cases are selected from the validation dataset to represent two scenarios at two frequencies. The first case is evaluated at a frequency of $200 \, \textrm{Hz}$, while the second case is evaluated at $800 \, \textrm{Hz}$ [70, 71]. For the implementation of the 2D SBM method, taken as the ground truth in this case, 266 and 249 uniformly distributed collocation points are placed along the boundary of the barrier for test cases 1 and 2, respectively, as shown in Fig. 9.

**Table 2:** Test cases for the T-shaped barrier

Case	Height [m]	Cap length [m]	Tortuosity [–]	Porosity [–]	AFR [Pa.s/m^2^]
1 (200 Hz)	2.9	0.56	2.5	0.22	6879
2 (800 Hz)	2.7	0.52	1.5	0.20	3478

**Fig. 10:**
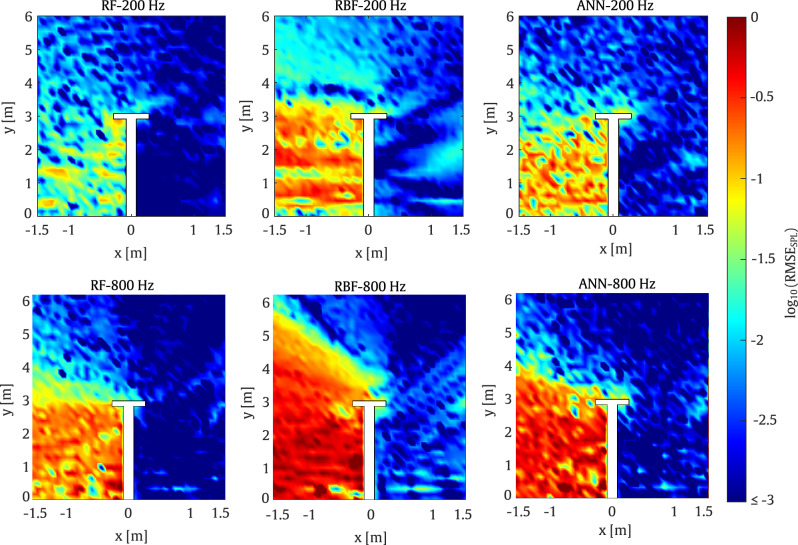
Comparison of SPL predictions from the surrogate models and the SBM at 200 Hz for the T-shaped barrier

As shown in Fig. 10, the results from the first example demonstrate good performance across the various surrogate models. On the one hand, the RF model exhibits very good agreement with the reference case, while the ANN models also provide acceptable accuracy. On the other hand, the RBF model shows greater discrepancies, particularly behind the barrier on the side opposite the source position.

**Fig. 11:**
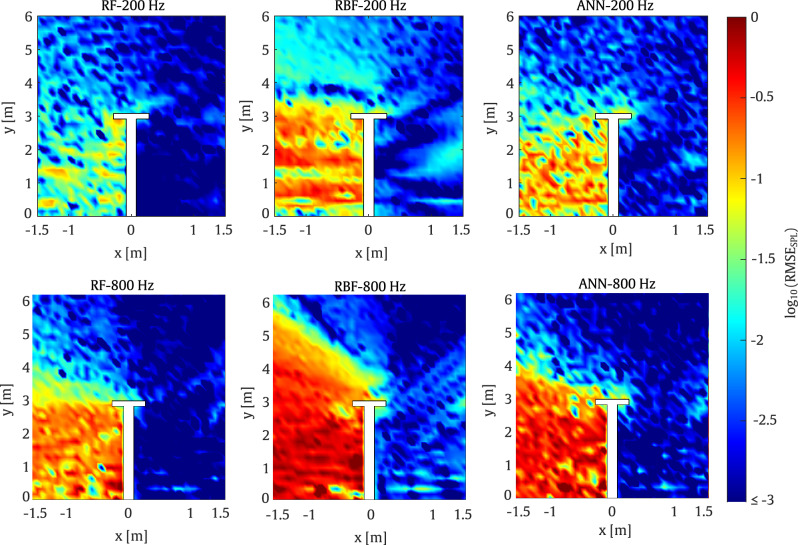
RMSE obtained by the surrogate models taking the SBM as the reference for a set of evaluation points at frequencies of 200 Hz and 800 Hz for the T-shaped barrier

This behaviour is also observed in Fig. 11 where the RMSE values for the RBF model reach up to 10% error over a substantial portion of the target domain. Thus, it can be concluded that the RBF model is the least accurate among the considered methods. In contrast, the RF model proves to be the most accurate, with errors of up to 6% observed only in a small region of the target domain.

In the second case, the models are evaluated at a frequency of $800 \, \textrm{Hz}$ using the parameters listed in Table 2. As shown in Fig. 11, the RMSE values for the ANN model are acceptable, with errors of up to 11% confined to a small region of the domain. It is worth noting that the large errors on the left side of the barrier are mainly due to the extremely low SPL in this area.

### Random forest with uncertain training data

In both single-objective Bayesian optimisation (SOBO) and MOBO, the uncertainty associated with surrogate models may misguide the search process, potentially leading to the selection of solutions with inferior true objective values. Consequently, relying exclusively on surrogate-based evaluations can result in suboptimal or even misleading decisions during the optimisation procedure. To address this limitation, it is essential to incorporate uncertainty information directly into the optimisation algorithm, thereby reducing the risk of erroneous solution selection and improving the robustness of the search process.

To account for uncertainty in the training data, the measurement error-filtered RF proposed in [72] is employed, which explicitly incorporates measurement errors into the RF modelling framework. In standard RF, all observations are treated as equally reliable because measurement errors are ignored, and the residual variance of the model is assumed to be constant. Let the true value of the target variable at location $s_i$ be denoted by

28 $$\begin{aligned} Y(s_i) = f\big (x(s_i)\,|\,\theta \big ) + \varepsilon (s_i), \end{aligned}$$

where *f* represents the RF model, $x(s_i)$ is the vector of covariates at $s_i$, $\theta$ denotes the model parameters, and $\varepsilon (s_i)$ is a stochastic residual capturing model error. Since the residual variance is assumed to be constant, the model is trained by minimising the sum of squared prediction errors, giving equal weight to all observations.

In contrast, the measurement-error filtered RF modifies this framework by incorporating measurement error variances into the loss function. Each observation is assigned a weight inversely proportional to the sum of the residual variance and its measurement error variance. Consequently, less reliable observations exert a weaker influence on model fitting. Estimation of the residual variance is achieved through an iterative procedure, as outlined in [72].

**Fig. 12:**
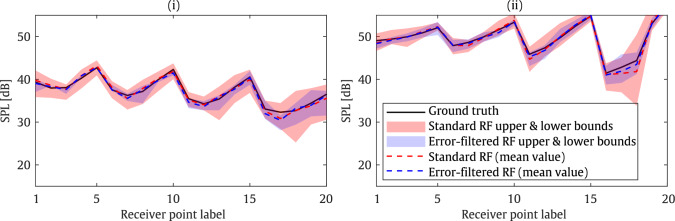
Comparison of the accuracy of case 1 (i) and case 2 (ii) against the ground truth, using both the standard RF model and the measurement error–filtered RF model

In this section, two cases are evaluated as shown in Table 3. To illustrate the impact of accounting for measurement errors, the results of cases 1 and 2 are compared against the ground truth in Fig. 12, within the framework of the T-shaped barrier configuration shown in Fig. 3 at a frequency of 200 Hz. The sound pressure level (SPL) was computed in the target zone indicated in Fig. 3, considering a total of 20 equally spaced receiver points. The lower and upper bounds were determined from the minimum and maximum predictions across all 20 trained meta-models, respectively. As shown, the standard RF yields a wider upper and lower bounds, whereas the measurement-error filtered RF provides predictions that align more closely with the ground truth. This, in turn, ensures the reliability and stability of the optimisation algorithm based on the RF model.

**Table 3:** Geometrical and material characteristics associated with both cases

Case	Height [m]	Cap length [m]	Tortuosity [–]	Porosity [–]	AFR [Pa.s/m^2^]
1	2.9	0.49	2.5	0.22	14038
2	2.26	0.54	2.9	0.17	14532

### Comparison of computational time

In this section, the computational efficiency of each surrogate model during training is presented in terms of normalised time, ranging from 0 to 1, using the equation below:

29 $$\begin{aligned} T_{normalised} = \frac{T - T_{\textrm{min}}}{T_{\text {max}} - T_{\textrm{min}}}, \end{aligned}$$

where $T_{\textrm{min}}$ and $T_{\textrm{max}}$ represent the minimum and maximum computational times among the surrogate models, respectively.

This comparison, alongside the numerical accuracy examples discussed in the previous cases, helps in selecting an appropriate surrogate model for the next steps, namely MOO. The results are evaluated for the first case of the T-shaped barrier, as described in the previous section, where 250 input samples for five selected parameters are used to train a surrogate model to predict acoustic wave propagation at 2,500 evaluation points in air at a frequency of 800 Hz. All three surrogate models were implemented in MATLAB and executed using the University of Birmingham’s primary computing facility, BlueBEAR.

**Fig. 13:**
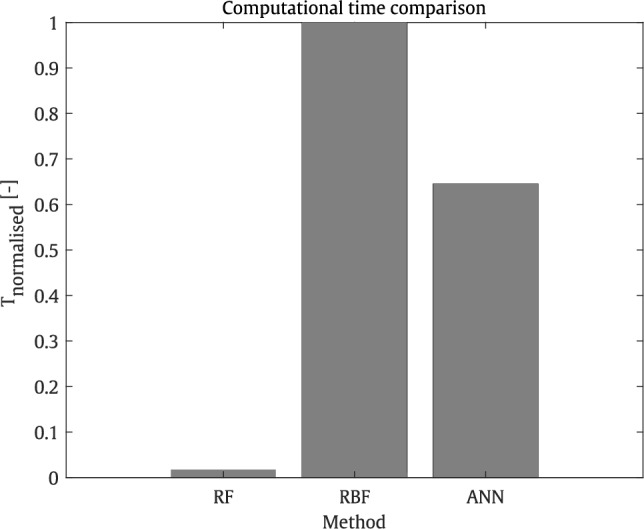
Comparison of the computational time presented by various surrogate models.

The results presented in Fig. 13 indicate that, based on the given configurations, the RF model represents the most efficient approach, whereas the RBF model is the most computationally expensive surrogate. Accordingly, their corresponding normalised computational times are set to zero and one for the RF and RBF models, respectively, denoted as $T_{\textrm{min}}$ and $T_{\textrm{max}}$ in Eq. (29). As illustrated, the RBF and ANN surrogate models exhibit substantially higher computational costs compared to the RF model. Consequently, to ensure a balanced trade-off between numerical accuracy and computational efficiency, the RF model has been selected for use in the MOO algorithm.

## Bayesian optimisation for porous acoustic barrier

Bayesian optimisation is an iterative two-stage procedure. In the data modelling stage, a probabilistic surrogate model is employed to approximate the objective function. In the guided search stage, an acquisition function is utilised to propose two candidate points in the parameter space for subsequent sampling. The acquisition function thereby balances the trade-off between exploring unexplored regions of the parameter space and exploiting those parameters for which observations are already available. This approach enables an efficient and reliable exploration of the parameter space in its entirety [73]. In this study, a Bayesian optimisation method is employed to optimise the porous acoustic barrier from both geometrical and material perspectives. The optimisation process is deployed through two case studies: a straight-walled barrier and a T-shaped barrier, both composed of porous materials, considering frequencies of $200 \, \textrm{Hz}$ and $800 \, \textrm{Hz}$. For each case, both MOBO and SOBO are presented. It is worth noting that, in order to render the optimal solutions more robust to the presence of Gaussian noise in the data, a Gaussian filter has been applied to the predictions of the RF model [74].

In the single-objective case, the aim is to minimise the acoustic pressure at a target zone while optimising the other five input parameters. The MOBO is employed to balance the trade-off between objective functions. Specifically, the goal is to minimise the barrier height and cap length while optimising the porous material properties, all with the aim of reducing the acoustic pressure at the target zone. The iterations continue for each optimisation case until the objective function value converges to a stable solution and further improvements in the objective function become negligible, ensuring an optimal balance between computational efficiency and solution accuracy.

For each case, the optimisation is repeated five times, and the final optimisation output is the average of these five repetitions. To ensure proper MOBO, normalisation is applied to the objective functions using Eq. (25). The locations of the source line and receiver zone where the average acoustic pressure is calculated can be found in Fig. 3.

### Single-objective optimisation

In the first optimisation example, a SOBO is performed to minimise the average acoustic pressure in the target zone with respect to all four parameters. The optimisation function is defined as:

30 $$\begin{aligned} \begin{aligned} \text {minimise} \quad&f(\textbf{x}) = \text {SPL}(\textbf{x}), \\ \text {where} \quad&\textbf{x} = \left[ \text {Porosity}, \, \text {AFR}, \, \text {Tortuosity}, \, \text {Height}, \, \text {Cap Length} \right] , \\ \text {subject to} \quad&\textbf{x} \in {\mathcal {X}}, \\&\text {For straight-walled barriers:} \quad \text {Cap Length} = 0, \\&\text {For T-shaped barriers:} \quad \text {Cap Length} > 0. \end{aligned} \end{aligned}$$

Here, $\textbf{x}$ denotes the vector of design parameters, while ${\mathcal {X}}$ corresponds to the design parameters’ bounds as indicated in Table 1. The results obtained for the straight-walled and T-shaped barriers are presented in the following sections.

#### Straight-walled barrier

**Fig. 14:**
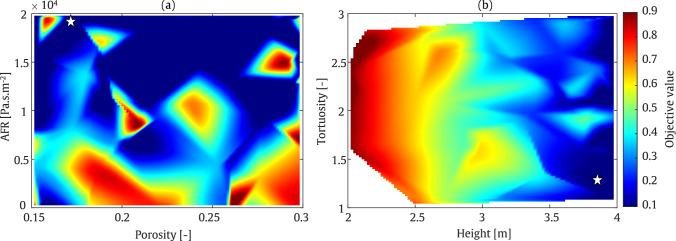
SOBO plot for straight-walled barrier at frequency of 200 Hz

**Fig. 15:**
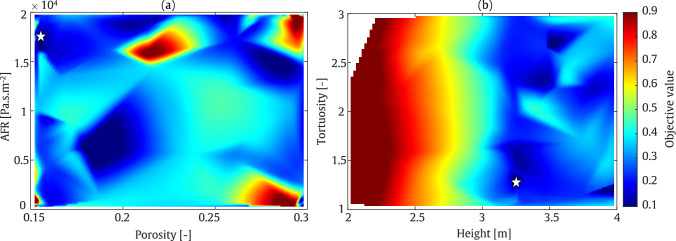
SOBO plot for straight-walled barrier at frequency of 800 Hz

To analyse the performance of the optimisation algorithm, colour map plots are presented for each considered frequency. The colour bars of these plots, which represent the objective function values as defined in Eq. (30), are normalised to the range 0.1–0.9 in order to ensure consistency across all optimisation colour maps in this study. This normalisation has been applied uniformly to all optimisation colour maps presented herein. These plots illustrate the optimisation of AFR and porosity in one plot, and height and tortuosity in another. As shown in Fig. 14, the minimum objective value is found at a porosity of approximately 0.17 and an AFR between 15,000 and 20,000 $\mathrm {Pa \cdot s/m^2}$. As expected, achieving the minimum SPL in the receiver zone requires a greater barrier height. Furthermore, tortuosity values between 1 and 1.5 demonstrate better performance as it enhances the absorption at low frequencies. At a frequency of 800 Hz, as illustrated in Fig. 15, higher barrier height and a lower tortuosity value, results in a lower objective function value.

These findings are quantified in Table 4. From this table, it can be seen that by optimising the four input parameters, the average IL at receiver zone, can reach to 34.1 dB and 44.8 dB at frequencies of 200 Hz and 800 Hz, respectively. The values at 200 Hz correspond to a sound-absorbing surface of the barrier, whereas the value at 800 Hz corresponds to a more reflective regime. Based on the material parameters provided in the table, the sound absorption coefficients at these frequencies are 0.2 at 200 Hz and 0.05 at 800 Hz, respectively.

Additionally, it should be noted that the white gaps observed during the optimisation procedure indicate that, owing to the guided nature of Bayesian optimisation, the algorithm selects only the most promising regions of the parameter space rather than evaluating every possible point.

**Table 4:** Final optimised values of the parameters using SOBO assessment: Straight-walled.

Frequency	Height [m]	Tortuosity [–]	Porosity [–]	AFR [Pa.s/m^2^]	IL [dB]
200 Hz	3.9	1.3	0.17	19757	34.1
800 Hz	3.2	1.2	0.16	17520	44.8

#### T-shaped barrier

**Fig. 16:**
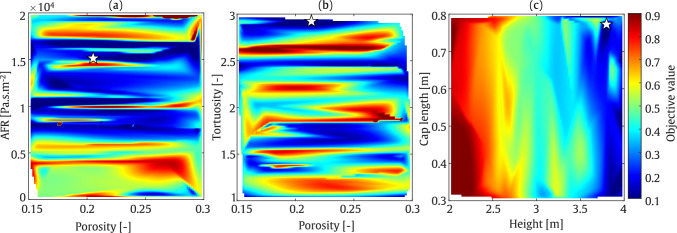
SOBO plot for T-shaped barrier at frequency of 200 Hz

**Fig. 17:**
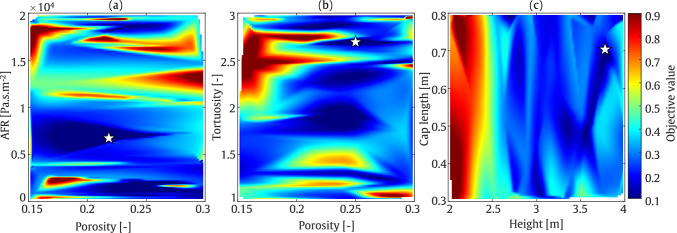
SOBO plot for T-shaped barrier at frequency of 800 Hz

In the second optimisation example, an SOBO is performed on a T-shaped barrier. The optimisation configuration is similar to that of the straight-walled case, with the addition of a barrier cap to the geometry. This added section results in output parameters that differ significantly from those of the straight-walled case. The colour map plots are presented in separate subfigures. Similar to Fig. 15, AFR is plotted against porosity, while porosity and tortuosity, as well as height and cap length, are plotted together. As shown in Fig. 16, the barrier height, cap length, tortuosity, and AFR of the porous material are optimised to nearly the highest values within the selected range, while porosity is set to mid-range values. At 800 Hz, as shown in Fig. 17, smaller AFR values are required to minimise the objective function.

These findings are quantified in Table 5, where the average IL in the receiver zone is reached to 39.6 dB and 51.3 dB at frequencies of 200 Hz and 800 Hz, respectively. As expected, the output IL for the T-shaped barrier is higher than that for the straight-walled barrier, particularly due to the significant role played by the barrier cap, the gain reaching 5 to 6 dB. The sound absorption values at 200 Hz and 800 Hz are approximately 0.4, indicating that the sound reduction may be attributed to the increased sound absorption properties of the barrier surface. As a result, the difference in sound reduction is likely due to the difference in sound wavelength alone.

**Table 5:** Final optimised values of the parameters using SOBO assessment: T-shaped.

Frequency	Height [m]	Cap length [m]	Tortuosity [–]	Porosity [–]	AFR [Pa.s/m^2^]	IL [dB]
200 Hz	3.7	0.76	2.9	0.21	15124	39.6
800 Hz	3.7	0.70	2.7	0.23	6962	51.3

### Multi-objective optimisation

The MOBO of porous acoustic barriers is the primary goal of this study. To reduce construction costs, the dimensions of the acoustic barrier should be minimised while maintaining its performance in mitigating acoustic wave propagation. In this context, two optimisation examples, configured similarly to the SOBO cases, are presented in this section. In addition, the optimisation function is formulated as:

31 $$\begin{aligned} \begin{aligned} \text {minimise} \quad&\textbf{f}(\textbf{x}) = \big [ \text {SPL}(\textbf{x}), \, \text {Height}, \, \text {Cap Length} \big ], \\ \text {where} \quad&\textbf{x} = \left[ \text {Porosity}, \, \text {AFR}, \, \text {Tortuosity}, \, \text {Height}, \, \text {Cap Length} \right] , \\ \text {subject to} \quad&\textbf{x} \in {\mathcal {X}}, \\&\text {For straight-walled barriers:} \quad \text {Cap Length} = 0, \\&\text {For T-shaped barriers:} \quad \text {Cap Length} > 0. \end{aligned} \end{aligned}$$

where $\textbf{x}$ is a vector of design parameters, and ${\mathcal {X}}$ represents the design parameters’ bounds as indicated in Table 1.

#### Straight-walled

**Fig. 18:**
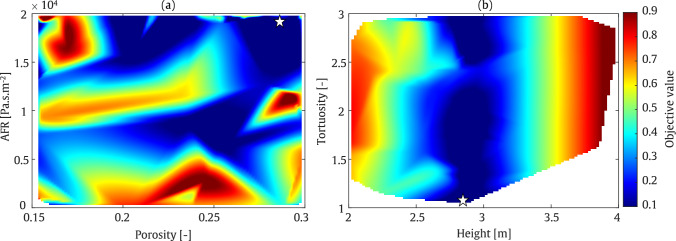
MOBO plot for straight-walled barrier at frequency of 200 Hz

**Fig. 19:**
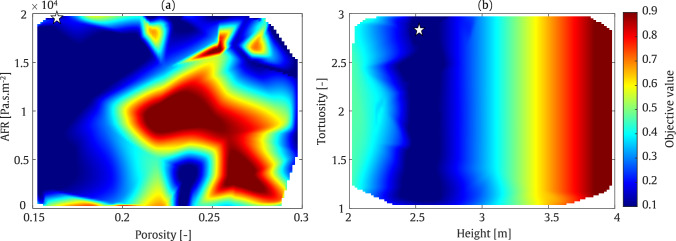
MOBO plot for straight-walled barrier at frequency of 800 Hz

The first example illustrates the MOBO of a straight-walled barrier. The output data exhibit behaviour significantly different from that obtained using the SOBO algorithm. As shown in Fig. 18, at a frequency of 200 Hz, the minimum objective function value is centred around a barrier height of approximately 3 m and a tortuosity between 1 and 1.5. The optimised values for material porosity and AFR are concentrated near their maximum ranges. This behaviour changes slightly at a frequency of 800 Hz. As depicted in Fig. 19, the minimum objective function values are located near the lower bounds of the barrier height. This aligns with the fact that a shorter wavelength at higher frequencies requires a lower barrier height. However, the porosity and AFR show multiple zones corresponding to the minimum objective function values. These qualitative findings are quantified in Table 6. As shown, the average IL in the target zone reaches 22.4 dB and 24.1 dB at frequencies of 200 Hz and 800 Hz, respectively. It should be noted that, based on the values presented in this table, the sound absorption levels at both frequencies are nearly identical. Additionally, the barrier height is minimised to 2.9 m at 200 Hz and 2.5 m at 800 Hz, respectively.

**Table 6:** Final optimised values of the parameters using MOBO assessment: Straight-walled

Frequency	Height [m]	Tortuosity [–]	Porosity [–]	AFR [Pa.s/m^2^]	IL [dB]
200 Hz	2.9	1.1	0.27	19652	22.4
800 Hz	2.5	2.8	0.16	19565	24.1

#### T-shaped barrier

**Fig. 20:**
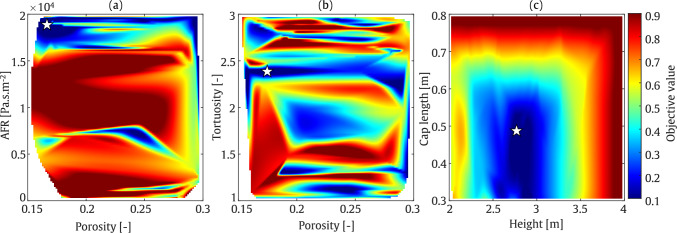
MOBO plot for T-shaped barrier at frequency of 200 Hz

The final optimisation example in this study concerns a T-shaped acoustic barrier. At a frequency of 200 Hz, as illustrated in Fig. 20, the minimum objective function value occurs at a barrier height of approximately 3 m and a cap length of 0.5 m. Various combinations of porosity and tortuosity parameters yield minimum objective function values, while larger AFR values lead to a further reduction in the objective function. In contrast to the value at 800 Hz, which corresponds to a low absorption value, indicating a rather reflective regime, this suggests that at this frequency, the sound reduction is primarily due to geometrical acoustics, rather than the addition of dissipation in the system.

**Fig. 21:**
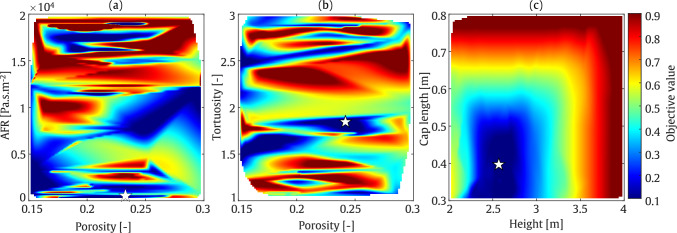
MOBO plot for T-shaped barrier at frequency of 800 Hz

At a frequency of 800 Hz, as shown in Fig. 21, a shorter barrier height and cap length are required to minimise the objective function. However, a large region in the colour map plot of porosity versus AFR, and porosity against tortuosity, exhibits low objective function values. As indicated in Table 7, the IL is approximately 29.5 dB and 45.1 dB in the range of SOBO at frequencies of 200 Hz and 800 Hz, respectively. These results correspond to a barrier height and cap length of 3 m and 0.5 m at 200 Hz, and 2.6 m and 0.4 m at 800 Hz.

It should be noted that, as expected, the T-shaped barrier provides superior acoustic performance compared to the straight-walled barrier. Specifically, an increase of up to 8 dB at 200 Hz and 20 dB at 800 Hz is observed when comparing the T-shaped and straight-walled cases.

**Table 7:** Final optimised values of the parameters using MOBO assessment: T-shaped

Frequency	Height [m]	Cap length [m]	Tortuosity [–]	Porosity [–]	AFR [Pa.s/m^2^]	IL [dB]
200 Hz	2.9	0.50	2.4	0.17	19712	29.5
800 Hz	2.6	0.40	1.85	0.24	235	45.1

#### Pareto front comparison

**Fig. 22:**
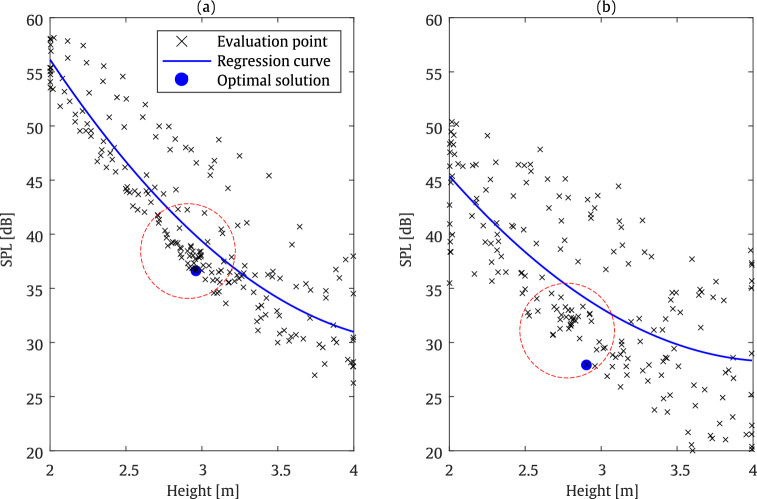
Comparison of the Pareto fronts for two objective functions in the MOBO algorithm, applied to **a** straight-walled and **b** T-shaped barriers

In this section, a comparison of the Pareto fronts is presented, considering two different objective functions: barrier height and SPL. These parameters are inherently contrasting, which may lead to conflict since increasing the barrier height generally leads to a reduction in SPL. In the MOBO framework adopted in this study, as discussed earlier, the aim is to minimise the barrier height and cap length (in the case of the T-shaped barrier), alongside SPL, as objective functions. As illustrated in Fig. 22, the goal is to achieve the trade-off between barrier height and SPL within the previously defined target zone. It should be noted that the results compared in this figure correspond to those obtained in Sects. 7.2.1 and 7.2.2, for the straight-walled and T-shaped barriers, respectively.

The comparison reveals that, firstly, an increase in barrier height results in a reduction of SPL. The regression curves highlight the Pareto front behaviour of the two objective functions. The dashed red circle indicates the search region where the Bayesian optimisation algorithm provides the most promising solutions. Furthermore, for the straight-walled barrier, the most favourable solutions are located at relatively higher barrier heights compared with the T-shaped barrier. This trend, also observed in previous evaluations, is due to the additional cap length in the T-shaped configuration. The optimal solutions are indicated with a blue marker, representing a balanced trade-off between the barrier height and the SPL value. Overall, this comparison underlines the importance of employing a MOBO approach to achieve a balanced trade-off between conflicting objectives, which cannot be attained using a SOBO strategy.

## Assessment of the performance of the optimised cases

**Fig. 23:**
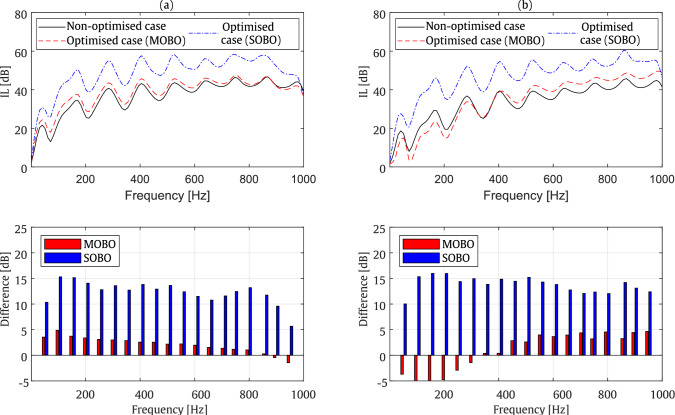
Comparison of SPL computed at evaluation receiver zone between the non-optimised case and optimised cases at 200 Hz (**a**) and 800 Hz (**b**), along with bar plots showing the differences between the cases in dB

To further highlight the differences between the optimised cases and the random validation case presented in Fig. 11 across the entire frequency range, additional results are provided using SBM approach in Fig. 23 for the T-shaped barrier case. These plots illustrate the IL differences between the optimised cases and the validation case at 50 Hz intervals. The comparison considers both optimised cases at 200 Hz and 800 Hz. In these plots, positive IL values indicate the extent of improvement achieved in the optimised case relative to the non-optimised case, while negative values represent a deterioration in the optimisation outcomes.

At both frequencies, SOBO demonstrates a significant improvement across the entire frequency range. In contrast to SOBO, MOBO mainly exhibits improvements around the tuned frequency. However, MOBO optimisation at 200 Hz results in slight improvements in IL values decreasing with frequency. Conversely, MOBO optimisation at 800 Hz leads to minor negative impacts of up to 5 dB at lower frequencies and improves with increasing frequency. These observations suggest that SOBO is better suited for broad-band optimisation, whereas MOBO is more appropriate when performance is only required near a specific, pre-defined frequency. If a global optimum across all frequencies is desired, SOBO offers a more robust solution. Alternatively, MOBO can be constrained or extended to cover a range of expected frequencies by modifying the objective function to incorporate performance across multiple frequency points, effectively turning it into a multi-frequency optimisation problem.

Overall, the results indicate that the proposed optimisation approach is highly effective in attenuating SPL across nearly the entire frequency range. However, MOBO does not achieve greater sound reduction compared to SOBO, though it does provide the optimal values for the barrier shape. In summary, this optimisation method offers a promising tool for enhancing the design of acoustic barriers, making it suitable for practical applications where a balanced trade-off between barrier dimensions and SPL mitigation is crucial. However, the results appear to be highly sensitive to the frequency range of optimisation.

## Graphical user interface application

To facilitate the broader adoption of the proposed metamodel, a Graphical User Interface (GUI) application was developed in MATLAB. The prediction and optimisation application is a standalone GUI program, organised with a tab-based interface. The main application consists of two primary sections: prediction and optimisation, as detailed below. The GUI application is available on https://github.com/HassanLiravi/AcousticBarrierGUI.

### Prediction GUI tab

The Prediction tab provides several inputs and options, which are detailed in the following.

**Fig. 24:**
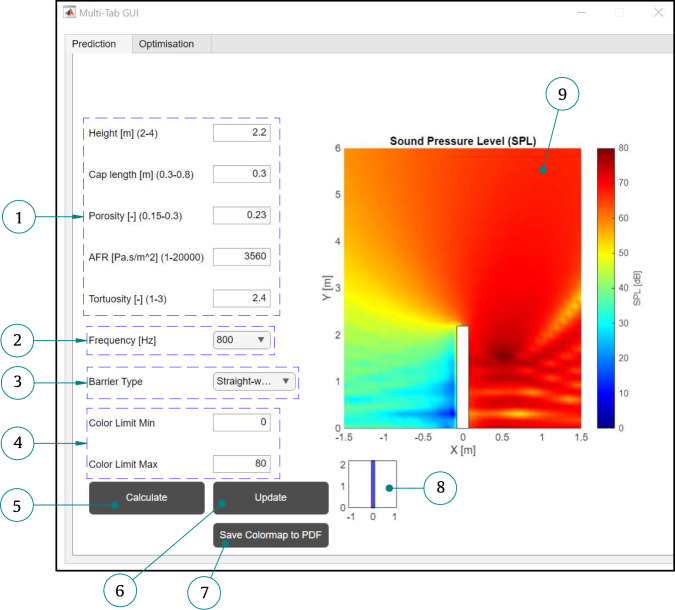
Prediction Tab GUI

1. **Design parameters (user input)**: The user can select the design parameter values in this section. The proposed framework allows the user to explore multiple scenarios by combining different design parameters.
2. **Input frequency selection**: The user can choose between the two frequencies considered in this study, namely 200 Hz and 800 Hz.
3. **Acoustic barrier type**: The GUI application incorporates both types of acoustic barriers analysed in this study, allowing users to choose between a T-shaped barrier and a straight-walled barrier.
4. **Colour bar limits**: The colour bar limits can be adjusted manually by setting the minimum and maximum values of the SPL in dB.
5. **Prediction process**: Once all the parameters mentioned above are defined, the user can initiate the prediction process by clicking the *Calculate* button. First, the trained models are loaded, followed by the prediction of SPL at 1,600 evaluation points distributed within the medium.
6. **Updating design parameters**: The user can adjust the design parameters using the *Update* button without the need to reload the trained models. In this case, the application directly generates predictions at the evaluation points. However, if modifications are made to other settings, such as frequency or barrier type, the predictions must be recalculated before proceeding.
7. **Saving results**: Once the predictions are completed and the colour map is displayed, the user can save the final graph in PDF format.
8. **Mini visualisation**: This feature enables the user to visualise the height and cap length (for the T-shaped barrier) in real time. Each time the height or cap length is modified, the user must also select the barrier type to update the visualisation.
9. **Colour map plot**: The colour map visualises the distribution of SPL around the selected barrier. The user can replicate the colour maps presented in this study for comparison and analysis (see Fig. 24).

### Optimisation GUI tab

The Optimisation tab comprises various inputs, which are defined below.

**Fig. 25:**
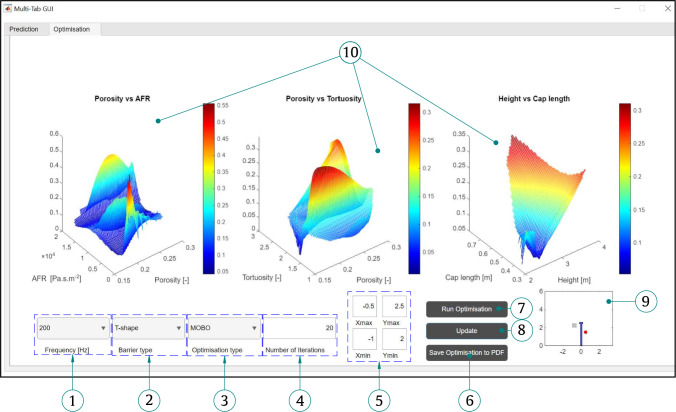
Optimisation Tab GUI

1. **Frequency selection**: Similar to the Prediction tab, the user can select either 200 Hz or 800 Hz as the frequency for the optimisation process.
2. **Barrier type**: The available barrier types, similar to those in the Prediction tab, include T-shaped and straight-walled barriers.
3. **Optimisation type**: The user can select the type of optimisation between MOBO and SOBO.
4. **Number of iterations**: The user can define the number of iterations for the optimisation process. This value significantly affects the computational time required for optimisation.
5. **Receiver zone definition**: The receiver zone can be specified by the user in this section. The minimum and maximum values of the receiver zone in the x and y directions can be set accordingly.
6. **Saving results**: The save button allows the user to save the optimisation graphs in PDF format.
7. **Run optimisation**: Once the input parameters are properly defined, the user can initiate the optimisation process by pressing this button. The application first loads the trained models before initiating the optimisation process, which can be monitored in real time through the mesh graphs.
8. **Updating parameters**: Similar to the Prediction tab, an *Update* button is embedded, allowing the user to modify the optimisation settings, barrier type, and receiver zone without reloading the trained models.
9. **Mini visualisation**: This feature enables the user to view the location of the source point (which is fixed in this GUI) and any changes in the receiver zone location instantly. To update the visualisation, the user must select the barrier type after modifying the receiver zone limits.
10. **Optimisation progress**: The optimisation process can be monitored in real time through these mesh graphs, which display the evolution of the design parameters (see Fig. 25).

## Conclusion

In this study, an accurate surrogate model is developed to expedite the simulation of acoustic barriers performance, enabling multi-objective optimisation of barrier design from both geometric and material perspectives. The model for predicting acoustic performance of barriers was initially developed in the basis of the fully meshless SBM. Then, a comprehensive numerical investigation of various surrogate models is conducted to evaluate their performance in terms of accuracy and computational time required for training. The surrogate model that offered the best balance of accuracy and efficiency was selected for subsequent SOBO and MOBO processes. The following key insights are derived from the numerical analyses presented:

- All developed surrogate models demonstrate satisfactory accuracy; however, the RF model outperforms the other approaches. The error delivered by the RBF approach is slightly higher in most test cases, ranking it as the least accurate model. Moreover, the LHS approach plays a crucial role in reducing the number of simulations required while maintaining high accuracy and reliability of the results. Additionally, the normalisation of input parameters enhances both the speed and accuracy of the learning process.
- The computational time required by the RF approach to train a surrogate model is among the lowest. In contrast, this value is significantly higher for the RBF and ANN models. Therefore, to achieve a balanced trade-off between numerical accuracy and computational efficiency, the RF model has been selected for the optimisation algorithm.
- The parametric study illustrates the sensitivity of different parameters. It indicates that porosity within the specified ranges of variation and tortuosity have the lowest impact on the IL, while height and cap length are the most influential parameters. Additionally, it is observed that, within the selected ranges, the barrier’s height and cap length, as well as porosity, have a direct relationship with the IL, whereas tortuosity and and AFR below 2500 $\mathrm {Pa.s/m^2}$ demonstrates an inverse relationship with the IL.
- Generally speaking, the T-shaped barrier exhibits enhanced noise attenuation capabilities compared to the straight-walled barrier which is an expected result in line with experimental and numerical results previously reported on the topic [4, 7].
- SOBO enhances the performance of the barrier across the entire frequency range, while MOBO results in a larger IL across a wide range of frequencies around the tuned frequency but not across the entire frequency range. Moreover, it may also degrade the barrier’s performance at frequencies far from the tuned one.
- MOBO is an excellent choice when the construction cost of the acoustic barrier is a concern. While the acoustic wavelength significantly influences the optimised parameters, the optimisation process, when tuned to a particular frequency, can result in improved acoustic performance of the barrier across a wide range of frequencies.

As a limitation, the optimisation algorithm must be carried out based on the target frequency, although it may also demonstrate deterioration at other frequencies, as evidenced by the results presented in this paper. Furthermore, the model could be improved and extended in future work to incorporate frequency in the LHS data. This enhancement would lead to a significantly faster training process and reduced storage requirements. While the applied Gaussian filter, together with the inherent capability of the RF model to handle noisy data, has resulted in an accurate and robust prediction model for optimisation purposes, a systematic investigation of robustness under varying noise levels would be valuable for future research. To conclude with a general remark, this study demonstrates the effectiveness of the proposed optimisation framework, which integrates the Bayesian optimisation algorithm with surrogate models constructed based on the fully meshless SBM approach. This framework efficiently navigates the trade-offs among multiple design objectives, offering a systematic methodology for optimising barrier designs. By doing so, it facilitates the creation of barriers that achieve an optimal balance between acoustic performance, material costs, and geometric constraints. The results underscore the potential of this approach to address complex multi-objective design challenges in a computationally efficient and practically relevant manner.

## Figures and Tables

**Fig. 26:**
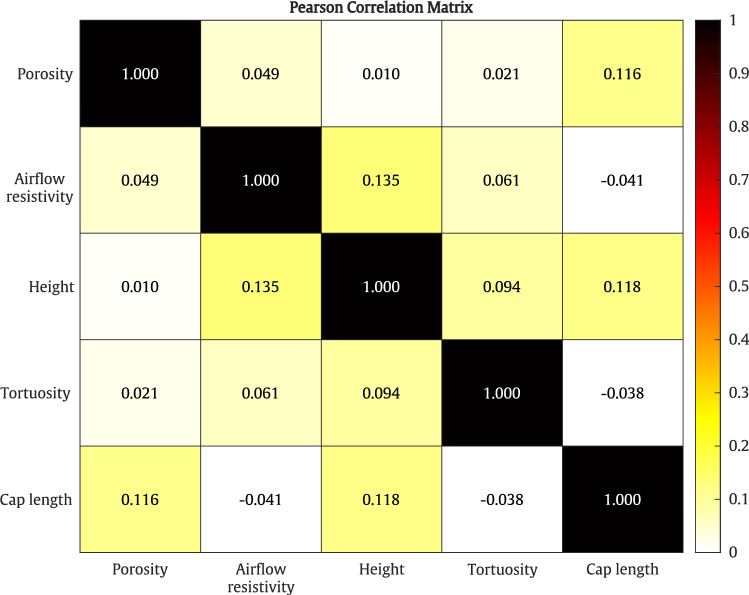
Pearson’s Correlation Matrix for the design variables

## Data Availability

No datasets were generated or analysed during the current study.

## Appendix A. Interaction strength between design variables

To assess the interdependence of the design variables, a correlation analysis is carried out in this section. Specifically, Pearson’s correlation coefficient analysis is employed. The Pearson correlation matrix, which describes the correlations among *N* variables, is generated from the training data previously introduced in MATLAB. This correlation matrix is a square, symmetric $N \times N$ (in this study, $5 \times 5$) matrix, with the (*i*, *j*)-th element equal to the correlation coefficient $r_{ij}$ between the *i*-th and *j*-th variables. A positive coefficient indicates that the two variables tend to increase or decrease simultaneously.

Here, the interaction effects among the five design variables used for the T-shaped barrier are assessed. The interaction strengths between the design variables are presented in Fig. 26. As shown, the interaction between barrier height and AFR exhibits the highest interaction index, while the interaction between cap length and barrier height ranks second. This assessment highlights the inter-correlation effects among the design variables.
